# Feature- versus rule-based generalization in rats, pigeons and humans

**DOI:** 10.1007/s10071-015-0895-8

**Published:** 2015-07-19

**Authors:** Elisa Maes, Guido De Filippo, Angus B Inkster, Stephen E. G. Lea, Jan De Houwer, Rudi D’Hooge, Tom Beckers, Andy J. Wills

**Affiliations:** KU Leuven, Tiensestraat 102, Box 3712, 3000 Leuven, Belgium; Università di Bologna, Via Zamboni 33, 40126 Bologna, Italy; Washington Singer Laboratories, University of Exeter, Exeter, EX4 4QG UK; Plymouth University, Drake Circus, Plymouth, PL4 8AA UK; Ghent University, Henri Dunantlaan 2, 9000 Ghent, Belgium; University of Amsterdam, Weesperplein 4, 1018XA Amsterdam, The Netherlands; KU Leuven, Tiensestraat 102, Box 3714, 3000 Leuven, Belgium

**Keywords:** Rats, Pigeons, Humans, Generalization, Rule-based, Associative models

## Abstract

Humans can spontaneously create rules that allow them to efficiently generalize what they have learned to novel situations. An enduring question is whether rule-based generalization is uniquely human or whether other animals can also abstract rules and apply them to novel situations. In recent years, there have been a number of high-profile claims that animals such as rats can learn rules. Most of those claims are quite weak because it is possible to demonstrate that simple associative systems (which do not learn rules) can account for the behavior in those tasks. Using a procedure that allows us to clearly distinguish feature-based from rule-based generalization (the Shanks–Darby procedure), we demonstrate that adult humans show rule-based generalization in this task, while generalization in rats and pigeons was based on featural overlap between stimuli. In brief, when learning that a stimulus made of two components (“AB”) predicts a different outcome than its elements (“A” and “B”), people spontaneously abstract an *opposites rule* and apply it to new stimuli (e.g., knowing that “C” and “D” predict one outcome, they will predict that “CD” predicts the opposite outcome). Rats and pigeons show the reverse behavior—they generalize what they have learned, but on the basis of similarity (e.g., “CD” is similar to “C” and “D”, so the same outcome is predicted for the compound stimulus as for the components). Genuinely rule-based behavior is observed in humans, but not in rats and pigeons, in the current procedure.

**Electronic supplementary material:**

The online version of this article (doi:10.1007/s10071-015-0895-8) contains supplementary material, which is available to authorized users.

## Introduction

Across the animal kingdom, organisms are capable of transferring what they have learned about a certain stimulus to novel stimuli. Generalizing newly acquired behavior is an important part of learning and allows the organism to respond quickly and adaptively. In the current article, we consider two types of generalization. First, generalization might be based on the perceptual features of stimuli. For example, when a tone (stimulus A) is followed by a shock, conditioned fear will generalize to another tone (stimulus B) to the extent that A and B are perceptually similar. If generalization is based on the perceptual features of stimuli, then it is said that generalization is feature-based. The second hypothesized type of generalization is rule-based. Humans can spontaneously create rules, which are not easily reducible to perceptual features, and which allow for efficient generalization of what is learned to novel situations (see below). The main question of this article is whether this rule-based route is uniquely human, as has been posited by some researchers (e.g., Penn et al. 2008).

Feature-based generalization is easily captured by association-formation theories, which state that when a stimulus (e.g., stimulus A) is presented, a set of representational elements is activated. Those elements might encode distinct features of stimulus A such as its pitch, duration, intensity, spatial location. When stimulus B is presented, some of the representational elements that are activated might be identical to those activated by stimulus A. The amount of generalization from stimulus A to stimulus B would then be a function of the number or proportion of elements A and B have in common (and/or the number or proportion of differences). The higher the featural overlap between A and B, the more generalization will be observed (e.g., Estes 1955; McLaren and Mackintosh 2000, 2002; Rescorla and Wagner 1972; Thorndike 1911; Tversky 1977). Other association-formation theories are based on variants of this general notion but incorporate additional assumptions about how exactly featural overlap is determined (e.g., Pearce 1994). In the current experiments, the latter theories make similar predictions to purely element-based accounts.

However, not all generalization outcomes observed in humans can be explained on the basis of featural similarity. Some instances of generalization seem instead to be rule-based and involving more complex cognitive mechanisms. In light of the enduring debate on the cognitive capacities of non-human animals, it has been suggested that rule-based generalization may be a uniquely human capacity (e.g., Penn et al. 2008). Hierarchies of cognitive ability have often been constructed on the basis of learning differences in abstract concepts and relational learning tasks (e.g., Wright 2010). However, as we will point out, much of this evidence has been inconclusive since viable associative explanations have not been ruled out convincingly.

Researchers have investigated whether pigeons can create arbitrary categories based on common consequences and then generalize within such categories. The general idea in those experiments is that if arbitrary categories of perceptually different stimuli are formed based on a common outcome (Vaughan 1988) or a common response (Wasserman et al. 1992), then changing the outcome or the required response for a subset of stimuli from one category should generalize to the other stimuli of the same category. Both Vaughan and Wasserman have observed such a generalization effect. However, if it is assumed that during generalization training, the presentation of a stimulus activates the representation of the response, which becomes associated with the new response, then association-formation models can explain generalization on the basis of common consequences (Wills et al. 2006).

A second line of research has focused on the ability to judge the relationship between two stimuli through an understanding of concepts such as same and different. It has been investigated whether pigeons (e.g., Blaisdell and Cook 2005; Katz and Wright 2006; Young and Wasserman 1997), rats (Wasserman et al. 2012), monkeys (e.g., Katz et al. 2002; Wright et al. 2003) and baboons (Fagot et al. 2001) can learn abstract concepts, such as same/different. Katz et al. (2007) have proposed several criteria that are important to rule out alternative explanations for abstract-concept learning. The procedure used by Blaisdell and Cook (2005) does not fulfill most criteria, e.g., due to questionable novelty of stimuli used during testing. Further, it seems that when multi-array stimuli are used [as in Fagot et al. 2001 (baboons), Wasserman et al. 2012 (rats), and Young and Wasserman 1997 (pigeons)], a simple measure of item variability can explain the behavior of the animals. Katz and Wright (2006) themselves have obtained evidence for same/different concept learning in pigeons, capuchin monkeys (Wright et al. 2003) and rhesus monkeys (Katz et al. 2002). However, it is possible that the pigeons in both the two-item same/different task (Katz and Wright 2006) and the matching-to-sample tasks (Bodily et al. 2008; Katz et al. 2008) performed the tasks by responding to recently seen items, because the target was always presented first followed by the choice options.

Rule-based generalization may also appear to underlie apparent analogical transfer, where the equivalence of the relationship between two sets of stimuli determines performance. Beckers and colleagues argued that rats can extract additivity rules and apply them to novel stimuli, shown as a modulation of the blocking effect by pretraining that provided information about the additivity of cues (Beckers et al. 2006). However, Haselgrove (2010) and Schmajuk and Kutlu (2010) suggested that the results of Beckers et al. (2006) can be accounted for by associative models (but see Guez and Stevenson 2011). Gillan and colleagues, reporting on the performance of the chimpanzee Sarah on both geometric and functional analogy problems, argued that she possessed the ability to reason on the basis of analogy (Gillan et al. 1981). In follow-up experiments, it was shown that Sarah could not only complete analogy problems, but could also construct analogies (Oden et al. 2001). However, as Penn et al. (2008) argue, replication and further examination of the underlying mechanisms are probably merited. Similar arguments apply to reports that an African grey parrot, Alex, can name the attribute on which a pair of objects are the same or different (Pepperberg 1987). Thus, a few observations suggest the presence of relational learning in animals, but further research is required.

Evidence from procedures developed to specifically investigate rule-based generalization seems to be mixed as well. While Preston (1986) did not find support for the generalization of a contextual rule, Murphy et al. (2008) did find that rats are able to generalize very basic sequential rules. On the other hand, several experiments point to the conclusion that pigeons are very efficient rote learners, but fail to learn overarching rules or concepts (Mackintosh 1988). The criterial-attribute procedure (Kemler Nelson 1984) and procedures based on the COVIS (COmpetition between Verbal and Implicit Systems; Ashby et al. 1998) framework, both originally aimed at investigating rule-based versus feature-based categorization in humans, have subsequently been used in comparative studies. Humans show rule-based generalization in the criterial-attribute procedure, while feature-based responding was observed in macaques (Couchman et al. 2010). However, recent work indicates that these conclusions may be an artifact of the inadequate analysis techniques employed (Wills et al. 2015) and comparative studies using less confounded techniques have found comparable levels of feature-based generalization responding across pigeons, squirrels and undergraduates (Wills et al. 2009). Similarly, in experiments based on the COVIS framework, it has been suggested that rule-based processes are available to humans (for a review see Ashby and Maddox 2005), and macaques (Smith et al. 2011), but not to pigeons (Smith et al. 2010). However, the evidence in humans has been challenged (e.g., Newell et al. 2011) and a number of issues have been raised with the results of the pigeon study (Edmunds et al. 2015). To complicate matters further, both in the criterial-attribute procedures and in comparative studies within the COVIS framework, the purportedly “rule-based” and “feature-based” behaviors also differ in the number of stimulus dimensions relevant for the different routes (Edmunds et al. 2015). For rule-based categorization, only one stimulus dimension is relevant, while for feature-based categorization multiple dimensions are relevant. This difference in dimensionality is problematic when considering the possibility that non-rule-based systems may have some mechanism of dimensional attention (e.g., Sutherland and Mackintosh 1971; Kruschke 1992). In other words, the seemingly rule-based responding in these procedures is explicable within an associative account under the assumption that participants attend to and learn about a subset of features (perhaps the most diagnostic features; Kruschke 1992). In consequence, those procedures do not allow us to clearly disentangle feature-based and rule-based mechanisms, so the controversy regarding the cognitive capacities of non-human animals remains.

In the human literature, there is one procedure for which nearly everyone on both sides of the debate agrees that rule-based generalization in this task is beyond simple associative accounts, the Shanks–Darby procedure. Shanks and Darby (1998), building on earlier work by Lachnit and Kimmel (1993), tested generalization after training on negative and positive patterning problems in human predictive learning. In negative patterning (NP) problems, stimuli A and B individually predict a certain outcome, but not when presented in compound (A+, B+, AB−). In positive patterning (PP) problems, a compound of two stimuli predicts an outcome, while the components do not (C−, D−, CD+). A general rule characterizes both patterning problems, namely compounds have the opposite outcome to their individual components (henceforth, an *opposites rule*). In the experiment of Shanks and Darby (1998), participants received training with complete positive and negative patterning problems, as well as incomplete positive and negative patterning problems. For example, in addition to training on A+, B+, AB−, C−, D− and CD+, participants saw I+ and J+, but not IJ and saw KL−, but not K or L. During testing, participants were confronted with the stimuli omitted during training. If generalization were feature-based, participants should predict the outcome on IJ trials, but not on K and L trials. A subset of participants, however, did not predict the outcome on IJ trials, but did predict the outcome on K and L trials—a pattern consistent with the opposites rule present in the training patterns. Participants who reached a high level of accuracy during training showed a generalization pattern consistent with an opposites rule, while participants that performed less well on the trained patterns showed a generalization pattern consistent with featural overlap.

Non-human animals have been shown to be capable of solving positive and negative patterning problems, even simultaneously (Dopson et al. 2011; Grand and Honey 2008; Harris et al. 2008; North and Price 1959; Pearce and George 2002). However, mastery of positive and negative patterning problems per se can be explained on the basis of associative mechanisms. For example, according to some association-formation theories, compounds generate configural cues, which emerge from the unique combination of A and B, and which in turn activate certain elements that are unique for the compound and are not shared with the components (Spence 1952). Negative patterning can then be solved by assuming that a configural cue, emerging from the combination of A and B, acquires strong inhibitory strength that cancels the combined excitatory strengths of the components A and B (Rescorla 1972). Thus, the evidence that animals can solve positive and negative patterning problems does not necessarily imply that they have also learned the underlying rule. Association-formation theories cannot, however, account for the rule-based generalization following successful simultaneous positive and negative patterning discrimination observed in humans. After all, when a new compound is presented for the first time, the configural cue has not yet gained any associative strength and therefore responding should depend entirely on generalization from the components to the compound (i.e., feature-based generalization).

Despite the clear superiority of the Shanks and Darby procedure over other procedures to test for rule-based generalization, to the best of our knowledge there are no reports of this paradigm being utilized with non-human animals. There is one report, by Davidson et al. (1993), where generalization of a negative patterning problem in rats was investigated, but generalization after simultaneous acquisition of a positive and negative patterning problems has never been tested in non-humans. Apparently rule-based generalization after mere negative patterning discrimination learning can be explained associatively, because low responding to the generalization compound could be explained by assuming that the inhibitory strength gained by the compound during the training phases generalized to the test compounds (on the assumption that compounds are more similar to other compounds than to non-compound stimuli). Our aim in the present studies, therefore, was to investigate whether non-human animals, rats (“Experiment 1A”) and pigeons (“Experiment 2A”), would be able to demonstrate generalization of negative and positive patterning rules. The conditions faced by the animals in the two experiments described here were quite different from the conditions ordinarily present in human studies of generalization of patterning rules. To allow for a fair comparison between the capacities of humans on the one hand and rats and pigeons on the other hand, we conducted two analog studies in humans that mimicked the conditions of the animal experiments as closely as possible (“Experiment 1B” and “Experiment 2B”).

## Experiment 1A: rats

In Experiment 1A, two groups of rats were trained on a negative patterning (A+, B+, AB−) and a positive patterning (C−, D−, CD+) problem simultaneously, in an operant conditioning procedure. One group was then trained on an incomplete positive patterning problem (E−, F−), while the other group was trained on an incomplete negative patterning problem (E+, F+). The crucial test consisted out of presentations of the novel compound (EF). According to feature-based models of generalization, responding to the novel compound should be similar to responding to its components (thus high for those animals for which E and F were reinforced and low for those animals for which E and F were not reinforced). If, on the other hand, rats were able to detect and apply the opposites rule, the reverse pattern should be observed, that is higher responding to the EF compound if E and F were not reinforced and vice versa.

### Methods

#### Subjects

The subjects were 24 experimentally naïve female Sprague–Dawley rats obtained from Janvier (France), with body weights ranging between 256 and 303 g at the start of training. Subjects were randomly assigned to one of the two groups (Ns = 12). The animals were pair housed in standard cages in a colony room that was illuminated from 8:00 a.m. to 8:00 p.m. The animals were allowed free access to food pellets (Sniff Spezialdiäten GmbH, Soest, Germany), whereas water availability was limited to 20 min per day following a progressive deprivation schedule initiated 1 week prior to the start of the study.

#### Apparatus

Eight standard operant chambers (34 cm length × 33 cm width × 33 cm height; Coulbourn Instruments, Leigh Valley, PA) housed in sound- and light-shielding cabinets (Coulbourn Instruments, Leigh Valley, PA) were used. All chambers had metal ceilings and side walls and clear Plexiglas front and back walls. The floor was made of stainless steel grids (0.5 cm in diameter). On one metal wall of each chamber, there was an operant lever, and adjacent to it was a recess (4 cm × 3 cm) centered 2 cm above the floor. A liquid dipper could deliver 0.04 cc of water into the bottom of the recess. Two speakers were mounted on each side wall. One was used to deliver a white noise at an intensity of approximately 73 dB(C). The second speaker was used to produce two tones, a low, pulsing tone [1000 Hz, 0.2 s on, 0.2 s off, ~79 dB(C)] or a high, complex tone [5000 Hz (0.6 s on, 0.1 s off) and 7000 Hz (0.6 s off, 0.1 s on), ~70 dB(C)]. A clicker was able to deliver a clicking sound, at an intensity of approximately 72 dB(C). A buzzer was used to deliver a buzzing sound, at an intensity of approximately 77 dB(C). The operation of a ventilation fan for each chamber contributed to the background level of noise that was approximately 65 dB(C). A light bulb, placed above the lever, was used to deliver a flashing light. Each chamber was illuminated by a dim house light placed on the opposite side of the light bulb. Those six different stimuli formed three sets of stimulus pairs: buzzer and flashing light (pair 1), low tone and house light turning off (pair 2) and high, complex tone and clicker (pair 3). Thus, two of the three compounds consisted of an auditory and a visual stimulus and one compound consisted of two auditory stimuli. All CSs were 30 s in duration. Water delivery was indicated by the onset of the white noise and the magazine light for 0.5 s.

#### Procedure

Before the beginning of the experiment, the three different stimulus pairs were assigned to the roles of AB, CD and EF in a counterbalanced fashion, yielding six counterbalancing types (see Table 1). Animals were run in three squads of eight rats balanced with respect to experimental condition and counterbalancing type. Each session was 62 min long.

**Table 1:** Design of Experiment 1A

*Group*	*Phase 1*
NP transfer	6 A+, 6 B+, 12 AB−, 6 C−, 6 D−, 12 CD+
PP transfer	6 A+, 6 B+, 12 AB−, 6 C−, 6 D−, 12 CD+
*Group*	*Phase 2*
NP transfer	2 A+, 2 B+, 4 AB−, 10 C−, 10 D−, 4 CD+, 8 E+, 8 F+
PP transfer	10 A+, 10 B+, 4 AB−, 2 C−, 2 D−, 4 CD+, 8 E−, 8 F−
*Group*	*Phase 3*
NP transfer	1 A+, 1 B+, 2 AB−, 2 C−, 2 D−, 2 CD+, 1 E+, 1 F+ / 2 EF− / 4 E−, 4 F−, 4 EF−
PP transfer	2 A+, 2 B+, 2 AB−, 1 C−, 1 D−, 2 CD+, 1 E−, 1 F− / 2 EF− / 4 E−, 4 F−, 4 EF−

The + represents 5-s access to 0.04 cc of water upon lever press, the − represents the absence of water; A/B, C/D and E/F represent buzzer/light off, clicker/low tone, and high tone/flashing light, counterbalanced. All stimulus presentations were 30 s in duration. The numbers represent the number of stimulus presentations per session. Commas separate interspersed trials, slashes separate different blocks of a phase that are not intermixed

##### Shaping

Standard procedures were used to train the rats to press the lever in order to obtain water. A fixed-time 120-s (FT-120-s) schedule of non-contingent water delivery was operated while the levers were retracted at the start of training; shaping ended on a variable interval 20-s (VI-20-s) schedule.

##### Phase 1

From days 1–27, rats received six presentations each of components A, B, C and D and twelve presentations each of compounds AB and CD (see Table 1). Stimuli A, B and the compound CD were followed by 0.04 cc of water accessible for 5 s upon lever press. Lever pressing during the components C and D and the compound AB was not reinforced. For the first five days, reinforcement was delivered on a continuous reinforcement (CRF) schedule. For the next 3 days (days 6–8), reinforcement was delivered on a variable ratio (VR) 2 schedule. Thereafter, reinforcement was delivered on a VR 4 schedule.

Trial order was semi-random so that no more than two trials of the same type and no more than four reinforced or unreinforced trials appeared in a row. The intertrial interval (ITI) ranged from 35 to 55 s with an average of 45 s. For the first 7 days of this phase, the lever was retracted during the ITI. After those 7 days, the lever was present throughout the whole session.

##### Phase 2

From days 28–36, rats continued to be trained on the negative and positive patterning problems, but additionally received eight presentations each of the generalization stimuli E and F. For the PP transfer group, lever pressing during presentation of the components E and F was not reinforced, while pressing to those components was reinforced for the NP transfer group. The number of A, B, C and D component trials was not equal between groups (see Table 1) in order to keep outcome frequency at 50 % overall as well as for presentations of components (20 reinforced, 20 unreinforced) and compounds (4 reinforced, 4 unreinforced).

##### Phase 3 (test phase)

On day 37, during the first part of the test phase all animals received presentations of the complete negative and positive patterns and the incomplete patterning stimuli as before. In the second part of this phase, the EF compound was presented twice, without reinforcement. In the third part, four unreinforced presentations of E and F were intermixed with another four unreinforced presentations of EF (see Table 1). This session lasted for 40 min.

### Data archiving

The session-level raw data are archived at www.willslab.co.uk/kulmaes1 with md5 checksum a4be13dfaa3476942874a930805a9198.^1^

### Results

**Fig. 1:**
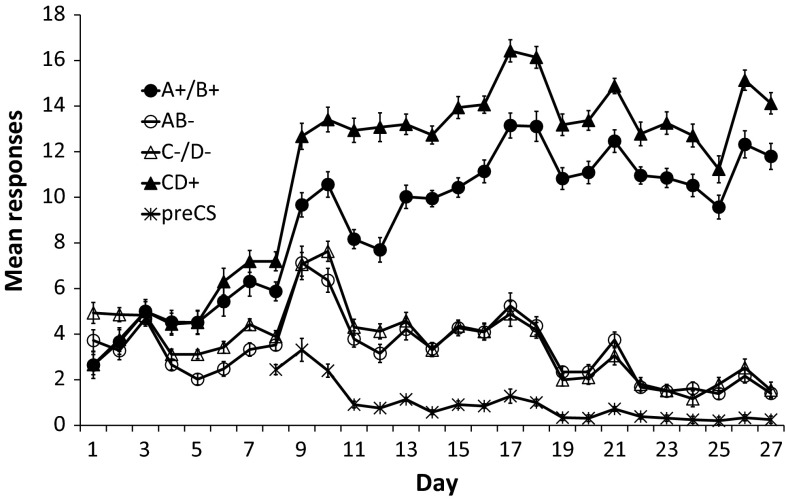
Mean number of responses over 30 s during reinforced and unreinforced components and compounds across the 27 days of Phase 1 training and mean number of responses over all 30-s prestimulus periods from the eighth day onwards. Error bars represent within-subject standard error of the mean for each stimulus as calculated by the SPSS plug-in of O’Brien and Cousineau (2014)

For the first phase, the mean number of responses (lever presses) made during the reinforced components A and B, the unreinforced components C and D, the reinforced compound CD and unreinforced compound AB are shown in Fig. 1. As can be seen, the mean number of responses made during the reinforced components and compound increased, while the number of responses made during the unreinforced components and compound decreased. Repeated-measures analysis of variance (ANOVA) with session and reinforcement (reinforced vs. unreinforced) as within-subject factors revealed an effect of reinforcement, *F*(1, 23) = 220.30, *p* < 0.01, $\eta_{\text{partial}}^{2}$ = 0.91, indicating an overall higher response rate to reinforced than unreinforced cues, a linear trend over sessions, *F*(1, 23) = 91.42, *p* < 0.01, $\eta_{\text{partial}}^{2}$ = 0.80, indicating an increasing response rate over training and an interaction between reinforcement and linear trend over sessions, *F*(1, 23) = 220.99, *p* < 0.01, $\eta_{\text{partial}}^{2}$ = 0.91, indicating an increase in discrimination between the reinforced and unreinforced stimuli over sessions. Follow-up analyses revealed that the response rate to the reinforced stimuli was higher than the response rate to the unreinforced stimuli from the fourth day of discrimination training onward, *t*(23) = 8.55, *p* < 0.01, 95 % confidence interval (CI) [1.21–1.99]. To investigate the apparent difference in speed of discrimination learning between NP and PP, an ANOVA with Session and Pattern (NP and PP) as within-subject factors was conducted on the difference between CS+ and CS− for each pattern. This analysis revealed an overall effect of Pattern, *F*(1, 23) = 12.62, *p* < 0.01, $\eta_{\text{partial}}^{2}$ = 0.35, a linear trend over sessions, *F*(1, 23) = 220.99, *p* < 0.01, $\eta_{\text{partial}}^{2}$ = 0.91, and an interaction between Pattern and linear trend over session, *F*(1, 23) = 6.79, *p* < 0.05, $\eta_{\text{partial}}^{2}$ = 0.23. These results indicate that the PP problem was learned more readily than the NP problem, as in previous reports (e.g., Harris et al. 2008, 2009). From the eighth day onwards, the lever was presented during the ITI and the number of responses during a 30-s prestimulus period was recorded. As can be seen in Fig. 1, the prestimulus response rate decreased over days.

**Fig. 2:**
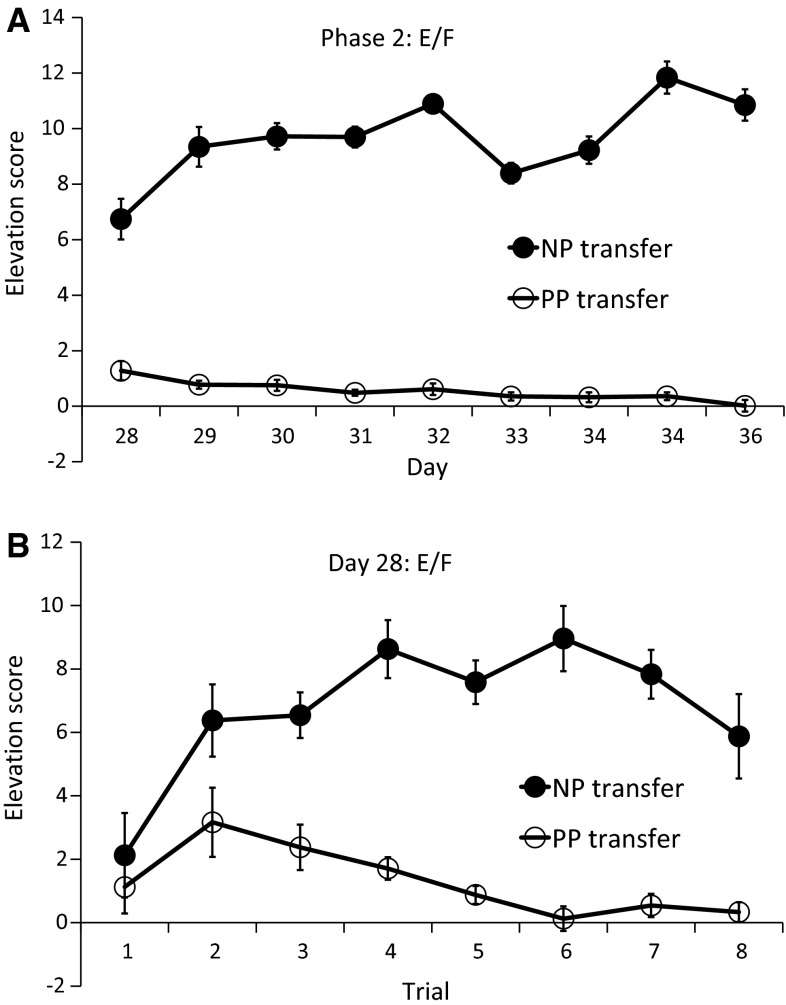
Mean elevation scores over 30 s for the generalization components E and F for groups NP transfer and PP transfer across the eight days of Phase 2 training (**a**) and across all trials of the first Phase 2 training day (**b**). *Error bars* represent within-subject standard error of the mean with group as between-subject factor as calculated by the SPSS plug-in of O’Brien and Cousineau (2014)

During the second phase, the lever was available throughout the whole session and an elevation score was calculated for each stimulus as the mean number of responses during each component or compound stimulus presentation minus the mean number of responses during the 30-s prestimulus interval for that specific stimulus. Responding to components E and F was higher in group NP transfer than in group PP transfer, as shown in Fig. 2, top panel. Since this difference was already apparent on the first day, we also examined responding on each trial of the first day (Fig. 2, bottom panel). Responding increased over trials for the NP transfer group, while responding decreased in the PP transfer group. An ANOVA with trial as within-subject factor and group as between-subject factor revealed an interaction between group and linear trend over trials, *F*(1, 22) = 8.87, *p* < 0.01, $\eta_{\text{partial}}^{2}$ = 0.29. Planned comparisons revealed a linear trend over trials in both groups, although only marginally significant for group NP transfer [NP transfer: *F*(1, 11) = 3.91, *p* = 0.07, $\eta_{\text{partial}}^{2}$ = 0.26; PP transfer: *F*(1, 11) = 7.93, *p* < 0.05, $\eta_{\text{partial}}^{2}$ = 0.42], suggesting that rats in the NP transfer group learned to respond to the new components and rats in the PP transfer group learned to not respond to those components. The average number of all 30-s pre-CS responses on this day was 0.35.

**Fig. 3:**
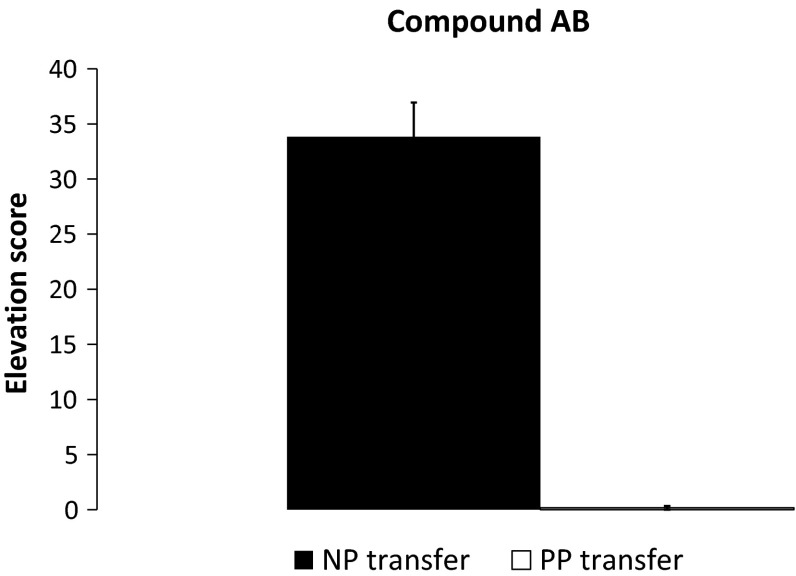
Mean elevation scores for the first 30-s presentation of the EF compound for groups NP transfer and PP transfer. *Error bars* represent standard error of the mean

During the actual test (Phase 3, parts 2 and 3), the EF compound was presented twice, unreinforced, followed by four unreinforced presentations of the components E and F, intermixed with four unreinforced presentations of the compound EF. The problem here is that extinction from the first two unreinforced presentations of EF might generalize to E and F (generalization of extinction effect), so that the response to E and F would be low. A lower response to E and F compared to EF might also be due to a higher chance to forget the E+/F+ training for E/F test trials than EF test trials. The crucial comparison is, therefore, the between-group difference in elevation score for the first presentation of EF. An independent *t* test revealed a higher elevation score for EF in the NP transfer group than in the PP transfer group *t*(11.06) = 10.82, *p* < 0.01, 95 % CI [26.82–40.51] (see Fig. 3). The average number of all 30-s pre-CS responses on this day was 0.54.

Finally, we determined the apparent generalization strategy (feature- vs. rule-based) for each individual rat. For animals in the PP transfer group, a standard deviation (SD) was calculated based on the responses to the unreinforced trials of the first part of Phase 3 (2 AB−, 1 C−, 1 D−, 1 E−, 1 F−). Rats in this group were classified as rule-based if the number of responses to the first presentation of EF was at least one SD above the mean number of responses to the first presentations of E and F. For animals in the NP transfer group, a standard deviation (SD) was calculated based on the responses to the reinforced trials of the first part of Phase 3 (1 A+, 1 B+, 2 CD+, 1 E+, 1 F+). Rats in the NP transfer group were classified as rule-based if the number of responses to the first presentation of EF was at least one SD below the mean number of responses to the first presentations of E and F. Using this criterion, none of the rats were classified as rule-based generalizers.

### Discussion

In this experiment, rats were trained on a positive and a negative patterning discrimination simultaneously. After 4 days of training, rats showed behavior consistent with having learned both the positive and negative patterning discriminations, which is considerably faster than published reports using purely Pavlovian training methods (Bussey et al. 2000; Harris et al. 2008, 2009). However, the use of an operant procedure in which the reinforcer is administered during the trial entails a potential problem. The first reinforcer delivered during a reinforced trial could serve as a cue for the availability of food during the remainder of the trial. This would lead to a high response rate on reinforced trials compared to unreinforced trials irrespective of any discrimination learning between the different stimuli (McDonald et al. 1997). There are two reasons for assuming that the rats did not rely solely on the presentation of the reinforcer to guide their behavior. Given that the reinforcer was delivered on a VR 4 schedule, on average four responses would be necessary to determine whether the trial would be reinforced or not. However, response rates to the unreinforced stimuli dropped below two by the end of Phase 1 (see Fig. 1). Moreover, high response rates to the EF compound were observed in the rats from the NP transfer group in the test phase, which was conducted under extinction (see Fig. 3), so that reinforcement could not serve as a cue for responding.

Despite the fact that the rats learned to solve the patterning problems quickly and reliably, generalization to the novel EF compound seemed to be fully feature-based. That is, elevation scores to the compound were higher in the NP transfer group than the PP transfer group. This is in sharp contrast with the human literature, where it has been shown that around 50 % of participants who learn to solve patterning problems generalize according to the opposites rule (Wills et al. 2011; see further analysis reported in Wills 2014).

A number of reasons might explain the discrepancy between the present results and the typical results in humans. The combination of auditory and visual cues might have made it more difficult for the rats to discern the underlying rule. Moreover, it might also limit generalization from an auditory–visual compound to an auditory–auditory compound. Also, by the time the generalization test was conducted, rats might have been overtrained on the patterning problems, which could have influenced retention of the rule. Another important note is that rats were trained on only one example each of positive and negative patterning, while humans are typically trained on at least two problems of each kind (Shanks and Darby 1998; Wills et al. 2011).

## Experiment 1B: humans

In Experiment 1A, rats did not demonstrate rule-based generalization after training on one negative and one positive patterning problem. In the rats’ defense, it is not clear from the human literature whether humans would demonstrate rule-based generalization under the conditions faced by the rats in Experiment 1A. Therefore, we conducted a very similar study with human participants. As in the rat study, an operant procedure using both auditory and visual stimuli was employed to train the participants on a negative and a positive pattern as well as an incomplete negative or positive pattern. Because humans learn this kind of discrimination much more quickly than rats, the procedure was compressed into a single session.

### Methods

#### Participants, apparatus and stimuli

Participants were 48 volunteers (8 male, mean age = 20.5 years) from KU Leuven. They received either partial course credit for an undergraduate psychology course or 4 euros for their participation in the experiment. Participants were tested individually in a quiet testing room using a PC connected to a 19-in. monitor and headphones and running Affect software (Spruyt et al. 2010). Four edited non-recognizable Microsoft Windows sounds served as auditory stimuli and two colored squares (blue and green) served as visual stimuli. In order to mimic the rat study, stimuli were paired such that two of the three compounds consisted of an auditory and a visual stimulus and one compound consisted of two auditory stimuli. Assignment of stimulus pairs to the roles of AB, CD and EF was counterbalanced within groups.

#### Procedure

The procedure of this experiment was developed through multiple pilot studies. On-screen instructions informed the participants that they had to press the space bar multiple times in order to gain golden coins and that the sounds they would hear and the images they would see would determine whether responding was rewarded or not. To impose a response cost, they were informed that a coin would be subtracted after every twentieth response. This information was repeated orally by the experimenter, after which a practice phase was initiated. At the start of the practice phase, the participants were informed that a butterfly was an example of an image that would lead to golden coins if they pressed the space bar and that the flower was an example of an image that would not lead to coins. A translation of the instructions given to the participants can be found in Online Resource 1 section I.

Throughout the experiment, the screen was black with a treasure chest in the right corner of the screen. The participant’s score was depicted on the chest in green. Below their score the text “best score: 341” was shown in order to motivate the participants. The value of this score was set at the beginning of the experiment and did not change during the experiment. The value of the score was chosen in such a way that it would be difficult, but not impossible to exceed it. After every twentieth response, “−1” appeared in the treasure chest in red and one point was subtracted from the participant’s total score. After a variable number of correct responses (i.e., bar presses during the CS+), a golden coin appeared on the screen and the participant’s score was increased by one point. Each stimulus was presented for 8 s with an ITI of 2 s.

During the practice phase, the butterfly and the flower were each presented five times, in a random order. During the first presentation of the butterfly, bar pressing was reinforced on a VR 3 schedule. The ratio was increased to 5 for the next presentation and was further increased to a VR 7 for the last three presentations. After the practice phase, the participants were informed that the experiment would start and they were asked to put the headphones on.

The design of the experiment is depicted in Table 2. In the first phase, participants were trained on a positive and a negative patterning discrimination, simultaneously. In the first part of Phase 1, participants received four presentations each of components A, B, C and D, and eight presentations each of compounds AB and CD. Bar presses made during the components A and B and the compound CD were reinforced on a VR 3 schedule, whereas bar pressing during the components C and D and the AB compound were not reinforced. In the second part of Phase 1, participants received three presentations each of the components and six presentations each of the compounds; the ratio schedule was increased to a VR 5. During the last part of Phase 1, participants received nine presentations each of the components and eighteen presentations each of the compounds, while the ratio schedule was increased to a VR 7. In total, participants received sixteen presentations of each component and thirty-two presentations of each compound in the first phase. Trial order was semi-random so that no more than two trials of the same type and no more than four reinforced or unreinforced trials appeared in a row.

**Table 2:** Design of Experiment 1B

*Group*	*Phase 1*
PP transfer	16 A+, 16 B+, 32 AB−, 16 C−, 16 D−, 32 CD+
NP transfer	16 A+, 16 B+, 32 AB−, 16 C−, 16 D−, 32 CD+
*Group*	*Phase 2*
PP transfer	8 A+, 8 B+, 3 AB−, 2 C−, 2 D−, 3 CD+, 6 E−, 6 F−
NP transfer	2 A+, 2 B+, 3 AB−, 8 C−, 8 D−, 3 CD+, 6 E+, 6 F+
*Group*	*Phase 3*
PP transfer	2 A, 2 B, 2 AB, 1 C, 1 D, 2 CD, 1 E, 1 F / 2 EF / 4 E, 4 F, 4 EF
NP transfer	1 A, 1 B, 2 AB, 2 C, 2 D, 2 CD, 1 E, 1 F / 2 EF / 4 E, 4 F, 4 EF

*A*–*F* represent four different auditory and two different visual stimuli; the + represents availability of reinforcement on a VR schedule; the − represents the absence of reinforcement. Commas separate interspersed trials, and slashes separate different blocks of a phase that are not intermixed

In the second phase, the generalization stimuli E and F were introduced while training on the negative and positive pattern was continued. As in the rat study, the number of A, B, C and D component trials was not equal between groups (see Table 2) in order to keep outcome frequency at 50 % overall and for presentations of components (19 reinforced, 19 unreinforced) and compounds (3 reinforced, 3 unreinforced).

After the second phase, new instructions appeared on the screen. The participants were now informed that they would no longer receive any feedback; however, the computer would keep track of their scores and they would see their total score at the end of the experiment. As with the rat study, participants first received trials containing previously encountered stimuli (see Table 2). In the second part, participants first received two presentations of the new compound EF, followed by another four presentations of EF intermixed with four presentations each of E and F.

### Data archiving

The trial-level raw data are archived at www.willslab.co.uk/kulmaes2 with md5 checksum 931a93e8e924c7d5116043680b30cd65.

### Results

**Fig. 4:**
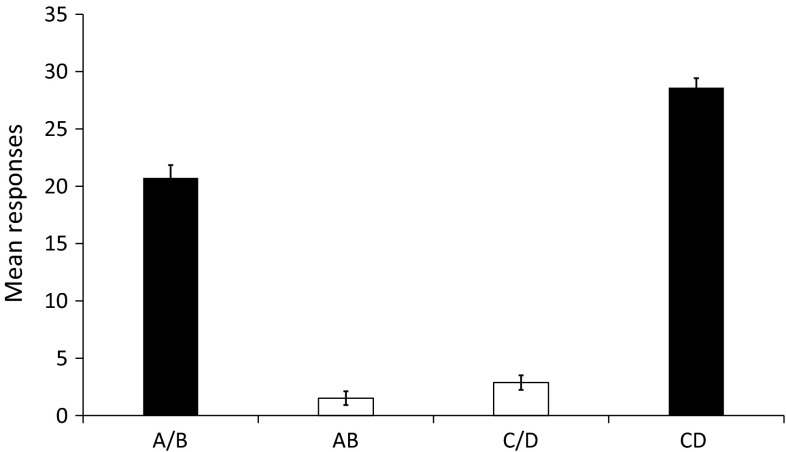
Mean number of responses during the last part of Phase 1 for reinforced components *A* and *B*, unreinforced compound *AB*, unreinforced components *C* and *D* and reinforced compound *CD*. *Error bars* represent within-subject standard error of the mean for each stimulus as calculated by the SPSS plug-in of O’Brien and Cousineau (2014)

To check participants’ mastery of the trained patterning discriminations, we analyzed the results of the last part of the first phase (the VR 7 part). The mean number of responses made during presentations of the reinforced components A and B, the unreinforced components C and D, the unreinforced compound AB and the reinforced compound CD are shown in Fig. 4. As can be seen, the mean number of responses during the reinforced components and compound is higher than the mean number of responses during the unreinforced components and compound. A *t* test confirmed that responding to the reinforced stimuli (mean 24.67) was higher than responding to the unreinforced stimuli (mean 2.19), *t*(47) = 22.29, *p* < 0.01, 95 % CI [20.45–24.50].

**Fig. 5:**
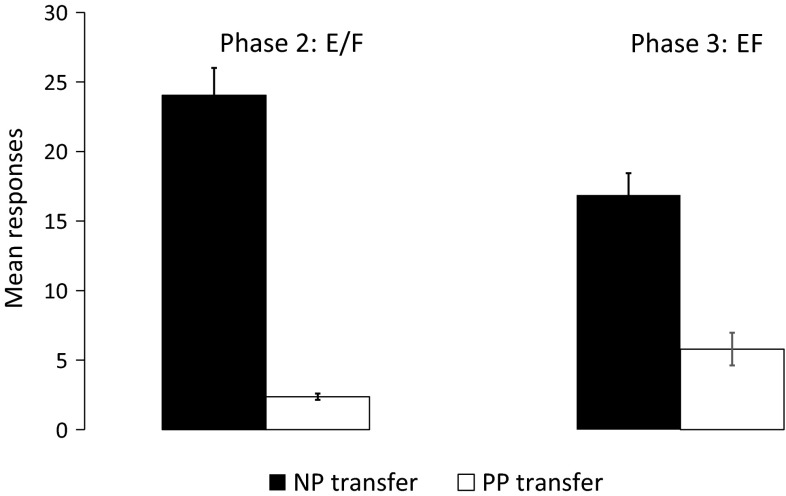
Mean number of responses during presentations of E and F during the last day of Phase 2 training (*left*) and mean number of responses during the first presentation of EF during Phase 3 training for NP transfer and PP transfer groups. *Error bars* represent between-subject standard error of the mean

During the second phase, responding to the new components E and F was higher in the NP transfer group than in the PP transfer group (see Fig. 5, left panel), *t*(23.60) = 10.92, *p* < 0.01, 95 % CI [17.57–25.77].

For the crucial test, we compared responding during the first presentation of EF between groups, as with the rat study. An independent *t* test revealed higher responding to EF in the NP transfer group than in the PP transfer group (see Fig. 5, right panel), *t*(42.67) = 4.00, *p* < 0.01, 95 % CI [5.50–16.67], suggesting feature-based generalization at the group level.

We also analyzed individual generalization strategies using the same criterion as for the rats. For participants in the PP transfer group, a SD was calculated based on the responses to the unreinforced trials of the first part of Phase 3 (2 AB−, 1 C−, 1 D−, 1 E−, 1 F−). Participants in this group were classified as rule-based if the number of responses to the first presentation of EF was at least one SD above the mean number of responses to the first presentations of E and F. For participants in the NP transfer group, a SD was calculated based on the responses to the reinforced trials of the first part of Phase 3 (1 A+, 1 B+, 2 CD+, 1 E+, 1 F+). Participants in the NP transfer group were classified as rule-based if the number of responses to the first presentation of EF was at least one SD below the mean number of responses to the first presentations of E and F. Using this criterion, thirteen participants from each group were categorized as rule-based.

As stated previously, none of the rats showed rule-based generalization, while 26 out of 48 human participants did. On a Chi-square contingency test, the human participants were significantly more likely to show rule-based generalization than the rats, *χ*^2^(1) = 20.35, *p* < 0.01.

### Discussion

The participants in this experiment were trained on one positive and one negative pattering problem using different auditory and visual stimuli in an operant conditioning paradigm. Participants in the PP transfer group were also trained on an incomplete positive patterning problem, and participants in the NP transfer group were also trained on an incomplete negative patterning problem. During the generalization test, two patterns seemed to emerge; some participants generalized based on featural overlap between the stimuli, while other participants generalized based on the opposites rule. To our knowledge, this is the first experiment to indicate that humans are capable of detecting the opposites rule in an operant conditioning procedure when trained on only one patterning problem of each kind and even when different stimulus modalities are used. The conditions faced by the participants in this experiment were rather similar to the conditions faced by the rats in Experiment 1A. In conclusion then, rule-learning appears more readily in humans than in rats, at least in the current procedure.

## Experiment 2A: pigeons

In Experiment 2A, pigeons were trained on two symmetrical patterning problems and four incomplete patterning problems in a go-left/go-right procedure using visual stimuli. During test, the pigeons were confronted with the novel compounds and the novel components. According to feature-based models of generalization, if the correct response for the components was the left response, then pigeons should also choose left when presented with the compound. If the reverse pattern should be observed, that is, pigeons choose left for the compound when the correct response to the components was right, it would indicate rule-based generalization.

### Methods

#### Subjects

The subjects were seven pigeons (*Columba livia*). They were housed in an indoor aviary and were transferred to individual cages on days when they were to be tested. After testing, they were weighed and given any supplementary feeding needed to maintain their weight at around 90 % of free feeding levels. On non-testing days, the pigeons remained in the aviary and were given a limited food supply there.

#### Apparatus

The experiment used seven identical operant conditioning chambers, measuring 710 × 505 × 435 mm. One long wall of each box included a 15-in. touch monitor, which consisted of an infrared touchscreen mounted in front of an LED computer display screen (ELO Touchsystems Inc Intellitouch, model 1547L). The bottom edge of the screen was 120 mm above the grid floor of the chamber. Two 2.8-W white houselights were mounted in the top corners of the operant panel above and to either side of the screen. Two recesses, each measuring 60 × 50 mm and giving access to grain hoppers when the hopper solenoids were activated, were located directly below the houselights and 40 mm above the grid floor of the chamber. The hoppers were illuminated by a 2.8-W white light when activated, and contained a 2:1 mixture of hemp seed and health conditioner, a highly preferred food for pigeons. White noise was played into the box from a loudspeaker located centrally below the touchscreen. The interior of the box could be observed by a video camera mounted on the side of the chamber. The chambers were housed in a darkened room together with other similar apparatus. Stimulus presentation and reinforcement contingencies for all chambers were controlled, and data recorded, by a customized PC (supplied by Quadvision Ltd, Dorset, UK) located in an adjacent laboratory area, with software written in Visual Basic using the Whisker control system (Cardinal and Aitken 2010).

#### Stimuli

**Fig. 6:**
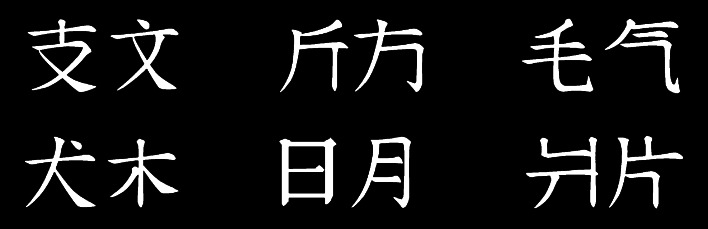
Six pairs of Chinese characters used in Experiments 2A and 2B

The stimuli comprised six pairs of Chinese characters, shown in Fig. 6. Each individual character was approximately 60 mm square and was displayed in white on a black background. For each bird, the character pairs were arbitrarily assigned to the six compound stimuli of the experimental design (AB, CD, EF, GH, IJ and KL, see Table 3). When presenting the component stimuli (e.g., A), a single appropriate character was shown. The two compound stimuli within any given patterning problem (e.g., AB and BA) differed only in the left–right placement of the two characters in the pair.

**Table 3:** Design of Experiment 2A and 2B

*Phase 1*						
Response 1	A, B					
Response 2	AB, BA					
*Phase 2*						
Response 1		CD, DC				
Response 2		C, D				
*Phase 3*						
Response 1	A, B	CD, DC				
Response 2	AB, BA	C, D				
*Phase 4*						
Response 1	A, B	CD, DC		GH, HG		K, L
Response 2	AB, BA	C, D	E, F		IJ, JI	
*Phase 5*						
Response 1	A, B	CD, DC	**EF**, **FE**	GH, HG	**I**, **J**	K, L
Response 2	AB, BA	C, D	E,F	**G**, **H**	IJ, JI	**KL**, **LK**

Responses 1 and 2 represent left or right response, counterbalanced; *A*–*K* represent different Chinese characters, counterbalanced; bold type indicates the critical test stimuli

#### Procedure

Standard procedures were used to train the pigeons to take food from either food hopper when it was operated. The pigeons were then trained to peck a 30-mm-diameter white circle located to the left of the touchscreen to obtain grain from the left hopper, and to peck a 30-mm-diameter white circle to the right of the touchscreen to obtain grain from the right hopper.

After this pretraining, birds were exposed to the Phase 1 go-left, go-right, training schedule (Table 3). Response 1 was left and Response 2 was right for four birds (*At*, *Ax*, *Mo*, *Ta*); for the other three birds (*Bw*, *Fe*, *He*), the assignments were reversed. For example, for bird *At* responses to the left were reinforced in the presence of stimulus A alone, and in the presence of stimulus B alone, while responses to the right were reinforced in the presence of stimulus compound AB and in the presence of stimulus compound BA.

At the beginning of each trial, a 30-mm-diameter white circle was presented centrally on the touchscreen. Two pecks on this circle replaced it with the target (e.g., AB), again centrally presented on the touchscreen. Two pecks to the centrally presented target replaced it with two copies of the stimulus; one copy was positioned on the left of the touchscreen, and the other on the right. One of those was the reinforced copy, and the other one was the unreinforced copy.

Pecks anywhere in a region centered around the reinforced copy, 200 pixels square for single-character stimuli or 400 × 200 pixels for two-character stimuli, were reinforced on a fixed-interval 3-s schedule with 2.5 s access to a 2:1 mixture of hemp seed and conditioner from the hopper nearer to the reinforced copy. Pecks to the other copy had no scheduled consequences. The trial was recorded as having a correct response if the first peck was to the reinforced copy. Reinforcement was followed by an ITI of between 3 and 6 s. Sessions consisted of 60 trials, with each trial type presented repeatedly and in random order. There were between two and five sessions per week.

Phase 1 training continued for each pigeon until it reached a criterion of 80 % correct in two consecutive sessions. Subsequent phases proceeded in a similar way, except that the trial types were of course different (see Table 3), and session length also varied slightly between phases to enable equal use of the different numbers of stimuli involved (Phases 2–4: 64 trials; Phase 5: 72 trials). Some birds failed to meet the learning criterion in some phases; for animal welfare reasons, these birds were progressed to the next phase after they reached a maximum number of sessions (at least 50 sessions, see Results for details).

### Data archiving

The trial-level raw data are archived at www.willslab.co.uk/exe3/ with md5 checksum af9a4c6f3703f180c5db9bd51019f549.

### Results and discussion

In Phase 1, learning of the patterning discrimination was generally rapid, with all but one bird taking between four and seven sessions to reach criterion (the remaining bird, *Ta*, reached criterion in 27 sessions). On transfer to the second patterning discrimination in Phase 2, all seven birds were below 50 % accuracy in the first session; this is consistent with the idea that the birds learned some kind of brightness or magnitude discrimination in Phase 1.

Learning of the Phase 2 patterning discrimination was slower than in Phase 1, with five birds taking between seven and fifteen sessions to reach criterion (*At*: 24 sessions; *Ta*: 37 sessions). Bird *At* died shortly after the end of Phase 2.

Phase 3 combined the patterning discriminations of Phases 1 and 2. Of the remaining six birds, three met criterion, taking 7 (Mo), 10 (Fe) and 43 (He) sessions to do so. One bird (Bw) progressed to Phase 4 after 22 sessions, having missed the criterion by a narrow margin (accuracies of 0.84 and 0.78 on the final two sessions). The remaining two birds did not reach criterion in the 60 sessions available, but their accuracy in the last two sessions was reasonably good (Ax: 0.67, 0.70; Ta: 0.75, 0.84). Accuracy across these last two sessions was significantly above chance for each of the six birds, min. *χ*^2^ = 18.00, *p* < 0.01.

Phase 4 added further compound and component trial types to Phase 3, but no further complete patterning problems (see Table 3), in preparation for the critical generalization tests at the beginning of Phase 5. Learning in Phase 4 was slow, with only one bird (Fe) reaching criterion within the 50–70 sessions available. Nevertheless, the birds’ accuracy in the last two sessions was reasonably good (Ax: 0.67, 0.72; Bw: 0.72, 0.64; He: 0.81, 0.77; Mo: 0.70, 0.89; Ta: 0.77, 0.64) and was significantly above chance for each of the six birds, min. *χ*^2^ = 16.53, *p* < 0.01.

Phase 5 completed the patterns of Phase 4 by the addition of novel test items. Accuracy exceeding 0.5 on these novel test items indicates rule-based generalization, while accuracy below 0.5 indicates feature-based generalization. As shown in Table 4, all six birds generalized on the basis of featural overlap rather than on the basis of the underlying rule (*p* = 0.03 on a two-tailed binomial test). All birds were above chance on the familiar stimuli (i.e., those also presented in Phase 4, see Table 4). Five of the six birds received 45–50 further sessions of training on Phase 5 (*Ta* received 10 further sessions). No bird reached criterion in Phase 5 in the time available.

**Table 4:** Results for Experiment 2A, Phase 5

Bird	Familiar	Novel
Ax	0.63	0.13
Bw	0.74	0.19
Fe	0.80	0.25
He	0.79	0.21
Mo	0.81	0.06
Ta	0.65	0.38

Accuracy for familiar stimuli and novel stimuli in Session 1

Accuracy below 0.5 on novel items indicates feature-based generalization

In summary, the pigeons found this task difficult but nevertheless demonstrated consistent patterns of responding to the novel test items. For all pigeons, generalization was feature-based, rather than rule-based.

## Experiment 2B: humans

Experiment 2B was, as closely as was practical, a human analog of Experiment 2A. Because humans learn this kind of discrimination much more quickly than pigeons, the procedure was compressed into a single session. A few changes to the procedure were made to facilitate this compression, see below. However, the phase structure (Table 3) and the stimuli were the same as in Experiment 2A, and the trial structure approximated that of Experiment 2A, modified to employ secondary reinforcement.

### Methods

#### Participants, apparatus and stimuli

Twenty-nine human adults (8 male, 19 female, 2 not recorded) were recruited through the School of Psychology’s participant panel at Plymouth University. Each was paid 8 GBP. The experiment was conducted using the E-prime package running on standard PCs with 19-in. monitors and standard keyboards. The stimuli were the same Chinese characters as used in Experiment 2A (see Fig. 6). Each participant experienced one of six different allocations of Chinese character pairs to compound stimuli, with allocations determined via a Latin Square design.

#### Procedure

The phase structure was the same as in Experiment 2A (see Table 3). For 15 participants, Response 1 was left and Response 2 was right; for the other 14 participants, the assignments were reversed. All participants were asked whether they were able to read Chinese characters (none were). They then received some basic instructions that described the structure of a single trial, but which did not reveal the phase structure and did not mention the word “rule” or any synonym thereof. The full instructions given to the participants can be found in Online Resource 1 section II.

Each participant was tested in a single session, with one block for the humans corresponding to one session for the pigeons. Humans were encouraged to rest briefly between blocks and had to press a key in order to proceed to the next block. Transitions between phases were not explicitly signaled. The learning criterion in Phases 1–3 was 0.80, the same as for the pigeons. In Phase 4, the criterion was lowered to 0.75, which was the mean last-block performance of the pigeons in Phase 4. The following changes, relative to the pigeon procedure, were made to keep the expected session length for humans below 1 h: (1) Humans had to pass the learning criterion for one block, rather than two, in order to proceed to the next phase, (2) humans progressed to the next phase after ten blocks if they had not met the criterion during that time (instead of 50+ sessions for the pigeons), (3) humans completed a single block of Phase 5.

At the beginning of each trial, a small fixation dot was presented in the center of the screen. Pressing the spacebar replaced the fixation dot with the stimulus (e.g., AB), again centrally presented. Pressing the spacebar again caused the centrally presented stimulus to be replaced by two copies of the stimulus; one copy was positioned on the left of the screen, and the other on the right. Participants pressed the “C” key to select the left-hand copy, and the “M” key to select the right-hand copy. If the participant’s response was correct, the stimuli were replaced by a centrally located yellow smiley face. Incorrect responses were followed by a blue sad face; 1000 ms after the participant’s response, the trial ended.

### Data archiving

The trial-level raw data are archived at www.willslab.co.uk/plym8/ with md5 checksum 33d885d9fe4d811d29367335372d3211.

### Results and discussion

Four of the 29 participants quit the experiment before completing Phase 3 and were excluded from further analysis. This 14 % non-completion rate matches the non-completion rate for the pigeons, although the reasons for non-completion were of course different.

For the remaining 25 people, learning in Phase 1 was fairly rapid, with participants taking an average of 1.52 blocks to reach criterion (SD = 0.92, range 1–4 blocks). Learning of the second patterning problem in Phase 2 was uniformly quick, with all participants reaching criterion in a single block. Note that pigeons found Phase 2 harder than Phase 1, while the reverse was true for humans. This difference in order of difficulty is consistent with the idea that people learn a patterning rule in Phase 1, which transfers positively to Phase 2, while pigeons learn a magnitude discrimination in Phase 1, which transfers negatively to Phase 2.

People also learned the Phase 3 combination of patterning problems rapidly, taking a mean of 1.60 blocks to reach criterion (SD = 1.15, range 1–5 blocks). Phase 4 added further compound and component trial types to Phase 3, but no further complete patterning problems (see Table 3). Two participants failed to meet criterion in Phase 4 within the ten blocks available, one participant approaching criterion in the final block, and one near chance. The remaining participants learned fairly rapidly, taking a mean of 2.22 blocks to reach criterion (SD = 1.78, range 1–8). All 25 participants progressed to Phase 5.

Phase 5 completed the patterns of Phase 4 by the addition of novel test items. Accuracy exceeding 0.5 on these novel test items indicates rule-based generalization, while accuracy below 0.5 indicates feature-based generalization. Table 5 shows accuracy on the novel test items for all 25 participants who completed the experiment. The majority of participants (16 of 25) generalized on the basis of the underlying rule. Critically, this was a significantly greater proportion of rule-based responders than had been observed in the pigeons, *χ*^2^ = 7.94, *p* < 0.01. Due to low expected values, Monte Carlo methods were employed in this test.^2^ The species difference remains significant if the humans failing the Phase 4 criterion are excluded from the analysis. It also remains significant under the conservative assumption that all four humans who did not complete the experiment would have shown feature-based generalization if they had.

**Table 5:** Results Experiment 2B

Human	Familiar	Novel	Human	Familiar	Novel
23	1.00	0.88	7	0.67	0.46
13	0.88	0.88	11	0.77	0.38
10	0.81	0.75	14	0.73	0.38
17	0.79	0.75	16	0.69	0.38
28	0.92	0.71	6	0.65	0.37
18	0.83	0.71	19	0.71	0.29
9	0.81	0.71	22	0.77	0.25
1	0.94	0.67	8	0.85	0.21
5	0.85	0.67	27	0.75	0.21
24	0.90	0.62			
25	0.73	0.62			
29	0.73	0.62			
5	0.75	0.62			
20	0.56	0.58			
26	0.48	0.58			
12	0.62	0.54			

Accuracy for familiar stimuli, and novel stimuli, in Experiment 2B, Phase 5

Accuracy above 0.5 on novel items indicates rule-based generalization (left-hand columns)

Accuracy below 0.5 indicates feature-based generalization (right-hand columns)

Note that the proportion of rule-based responders did not significantly exceed the proportion of feature-based responders, *χ*^2^(1) = 1.96, *p* = 0.16. Such an effect would not be expected given the 75 % criterion in Phase 4. Previous studies using the Shanks–Darby procedure suggest that terminal training accuracies of at least 90 % are required to ensure a significant group-level preference for rule-based generalization in humans (Shanks and Darby 1998; Wills et al. 2011). In the current experiment, the criterion was set at a lower level to approximate the level of performance observed in the pigeons.

In summary, all pigeons in Experiment 2A showed feature-based generalization, while the majority of humans in Experiment 2B showed rule-based generalization. Rule-learning again appears more readily in humans than in non-humans, at least in the current procedures.

## General discussion

In the experiments described above, rats, pigeons and humans were trained on one instance each of two symmetrical patterning problems. In Experiments 1A and 1B, rats and humans were then trained on one incomplete pattern, either negative or positive, while in Experiments 2A and 2B, pigeons and humans were trained on four incomplete patterns. During test, responding to the complementary stimuli was recorded. All animals (including humans) were able to master both patterning problems. However, despite mastery of the problems, generalization was feature-based in each and every one of the rat and pigeon subjects, while a majority of the human participants showed rule-based generalization. Our results suggest that seemingly rule-based behavior in non-human animals may be explained on the basis of simpler cognitive mechanisms and that non-human animals are less prone to exhibit rule-based generalization than humans under similar circumstances.

There are some important differences in procedure between Experiments 1A and 1B on the one hand and 2A and 2B on the other hand. The rats did seem to learn the patterning problems quite rapidly compared to the pigeons. This might be due to a difference in go/no-go and go-left/go-right procedures, where the latter are possibly more difficult. More likely, the difference is due to the difference in similarity between the stimuli used in the rat and human–rat analog on the one hand and the pigeon and human–pigeon analog on the other hand. On almost any measure, e.g., A and AB are more similar in the pigeon experiment than the rat experiment. Then again, the go-left/go-right procedure has a clear advantage over the go/no-go task, with the former allowing clearer investigation of generalization from E and F. In the rat study, low levels of responding to EF are consistent with feature-based generalization but are also consistent with the animals not having learned anything about E and F. The trial-based analysis of Phase 2 shows a decrease in responses to E− and F− over trials, suggesting that the rats did learn not to respond to E and F, but in a go-left, go-right procedure, those two options can be distinguished more clearly (with a lack of learning yielding chance performance and feature-based generalization yielding a preference for one side over the other). Another advantage of the pigeon and human–pigeon analog over the other two experiments is that the former allowed tests of both generalization to components and to compounds. This would have been important if rule-based generalization had been observed in the rats, because the model of Verguts and Fias (2009), which is the only extant associative model able to provide a partial explanation of rule-based generalization of an opposites rule, can explain seemingly rule-based generalization to compounds only, not to elements. Thus, if rule-based generalization in the rat study would have been found, we would not have been able to completely exclude an associative explanation (although it is a matter of debate whether the Verguts-Fias model counts as an associative model in the normal sense, see Wills et al. 2011, for further discussion). Another remark concerns the difference between the fixed amount of training used in Experiments 1A and 1B and the variable amount of training based on performance used in Experiments 2A and 2B. Theoretically, it is possible that there was a difference in the extent to which the rats in Experiment 1A were overtrained compared to the humans in Experiment 1B, which might explain the difference in the degree of rule-based generalization between rats and humans. However, this cannot be said about Experiments 2A and 2B, because the subjects in both experiments were trained to criterion. Finally, in Experiments 1B and 2B, different reinforcers were used (accumulation of points vs. happy/sad faces), which were both effective in motivating and reinforcing the participants. The diversity of the designs probably increases the generality of our findings.

The goal of the present experiments was to investigate whether non-human animals would be capable of rule-use, a capacity recently claimed to be uniquely human (Penn et al. 2008). While evidence for other human-like cognitive processes such as abstract concept and relational learning has been scarce at best (see “Introduction” section), the results described in the current paper are indicative of an absence of rule-based learning in rats and pigeons. However, it might be premature to conclude that rule-based processes are indeed absent in those two species.

For one thing, the observed difference between rats and pigeons on the one hand and humans on the other could perhaps be due to a difference in speed of learning. It is possible that non-humans when learning are pushed by the difficulty of the task into adopting a configural strategy, which is unconducive to rule extraction. Humans, who learn more rapidly, may not be forced down this route and may instead apply an elemental strategy which is conducive to rule extraction. However, there are at least two problems with this explanation. First, empirically, we do not find much support for a relation between speed of learning and rule-based generalization in our data; e.g., in Experiment 2B, there was no correlation between total number of training blocks and degree of rule-based generalization (*r* = −0.18, *t*(23) < 1, *p* = 0.38). Second, theoretically, only a hyper-configural strategy, i.e., with no or very little feature-based generalization between the compound and its components, would reduce inference and thus decrease task difficulty. However, this hyper-configural strategy should prevent all generalization at test, be it rule-based or feature-based, while the test results clearly indicate feature-based generalization in rats and pigeons.

Yet, while rats and pigeons did not seem to extract rules in the current procedure, it cannot be excluded that those animals would show rule-based behavior under different circumstances. Important here is to note that opposites rule generalization is probably quite challenging. Indeed, only about half of the adult participants who master the patterning problems show rule-based behavior (Wills et al. 2011; see further analysis reported in Wills 2014), and it has been shown that under cognitive load even participants that master the patterning problems show feature-based generalization (Wills et al. 2011). If one makes the minimal assumption that rats and pigeons have more restricted cognitive capacities than humans (even if not qualitatively different), detection of the opposites rule in patterning problems might prove to be too difficult, while not excluding that rats and pigeons are capable of rule-based generalization when dealing with simpler rules. A valid reason for assuming that rats, and by extension pigeons, might show rule-based behavior in other tasks is the observation that rats are capable of generalizing sequential rules (see “Introduction” section; Murphy et al. 2008). Sequential rules are probably easier to detect and apply to a new set of stimuli. Children from the age of 7 months onward will generalize on the basis of rules in a task similar to the one employed by Murphy and colleagues (Marcus 1999). It would, therefore, be interesting to investigate whether the application of simpler rules that emerge relatively early in human life can be demonstrated in animals.

In addition, Katz, Wright et al. have argued that, in order to investigate the presence or absence of a certain cognitive capacity, it is important to test animals repeatedly, providing an increasing number of examples (Wright 2010). In an experiment with pigeons, it was shown that pigeons do not show same/different discrimination after training with only a few examples, whereas such capacity does emerge after training with an extensive amount of examples (Bodily et al. 2008; Katz and Wright 2006). Katz et al. further demonstrated that the number of examples at the start of training matters as well. When training commenced with only a small number of examples, carryover effects hampered the performance of pigeons during generalization testing, but when pigeons received training with an extensive amount of examples from the beginning, same/different generalization was observed on the first test session (Nakamura et al. 2009). Given that relational learning in monkeys emerged faster, thus after fewer examples, than in pigeons (Wright and Katz 2006), it is possible that rule-based generalization in the Shanks–Darby task might be observed when animals receive training on multiple examples. Certainly, when considering that humans have much more experience with the concept of oppositeness and rule-use in general than animals, it might be worthwhile to investigate whether opposites rule generalization would emerge in rats and pigeons with extended experience.

## Electronic supplementary material

Supplementary material 1 (DOCX 19 kb)

## Footnotes

1 Publication of an MD5 checksum allows the reader to independently confirm that the raw data in the archive are unchanged.

2 Specifically, we used the Chi-square test function in the *stats* package of the R environment (R Core Team 2014), with 10^6^ iterations. Chi-square test uses Patefield’s (1981) algorithm.
