# Supplementary material for: Feature- versus rule-based generalization in rats, pigeons and humans
Source: Anim Cogn. 2015 Jul 19;18(6):1267–84. doi: 10.1007/s10071-015-0895-8 (PMC4607717; doi:10.1007/s10071-015-0895-8)
Supplement: Supplementary file 1 — Supplementary material 1 (DOCX 19 kb) [file 10071_2015_895_MOESM1_ESM.docx]

Animal Cognition

Feature- versus rule-based generalization in rats, pigeons and humans

Elisa Maes^1^, Guido De Filippo^2,3^, Angus Inkster^4^, Stephen E.G. Lea^3^, Jan De Houwer^5^, Rudi D’Hooge^1^, Tom Beckers^1,6^ and Andy J. Wills^4^

^1^ KU Leuven, Belgium

^2^Università di Bologna, Italy

^3^University of Exeter, UK

^4^Plymouth University, UK

^5^Ghent University, Belgium

^6^University of Amsterdam, The Netherlands

Corresponding authors:

Tom Beckers

TEL: +32 1632 6134

FAX: +32 16 3 26099

E-mail: [tom.beckers@ppw.kuleuven.be](mailto:tom.beckers@ppw.kuleuven.be)

Andy Wills

TEL: +44 1752 584816

E-mail: andy@willslab.co.uk

## **Online Resource 1**

### **Instructions Experiment 1B [translated from Dutch]**

*Instructions part 1*

The goal of this game is to collect golden coins. At this moment, the highest score is 341. Can you do better?

You can collect coins by pressing the space bar. During the game, you will hear different sounds and see various images, sometimes alone, sometimes in combination. You will notice that those images and sounds determine whether you obtain coins for pressing the space bar. You will need to press MULTIPLE TIMES in order to gain A LOT OF coins.

For every twentieth press, a coin will disappear from your treasure chest. So learn as fast as possible which images, sounds and combinations yield golden coins, this is to only way to beat the best score.

Good luck!

Inform the experimenter when you’re done reading the instructions.

*Instructions part 2*

Now comes an example.

The butterfly is an example of an image that will yield golden coins if you press the space bar multiple times. The flower is an example of an image that will not yield a reward.

Press enter to start the example

*Instructions part 3*

[These instructions are provided after the practice phase]

This is the end of the example.

Your score will be reset to zero. Can you beat the best score?

Press enter to start

*Instructions part 4*

[These instructions are provided before the start of the training phase]

From now on, you will not see whether you gain or lose coins. Your score will disappear from the screen as well, but the amount of coins you gain will be recorded! Other things will remain as before.

At the end, you will see how many points you have won in total.

Press enter after reading the instructions.

### **Instructions Experiment 2B**

This study uses Chinese characters as a form of abstract picture.

If you are able to read Kanji script, please tell the experimenter now.

If you can't read Kanji - great! You don't need to be able to in order to do this study.

Welcome to the experiment!

In this study, you will be shown some abstract pictures.

You job is to learn which group each belongs to.

For each picture, the correct group response will be one of two keys.

The first thing you will seen is a small dot on the screen.

This tells you where the picture is going to appear.

Press the SPACE bar when you're ready to see the picture.

Take a look at the picture.

When you are ready to make your response, press the SPACE bar again.

When you press the SPACE bar for a second time,

the picture will disappear from the centre of the screen,

and re-appear to the left and right of the screen.

Your response should now be to pick one of these two identical pictures.

In order to pick the left-hand picture (Group 1), press the 'C' key.

In order to pick the right-hand picture (Group 2), press the 'M' key.

At first you will be guessing. However, after each response,

you will see either a smiley face (your response was correct)

or a sad face (your response was wrong).

Your task is to learn from this feedback to respond correctly.

Although this may be difficult at first, there are no tricks,

and it is possible to score 100%. You should aim to do this.

Press the SPACE bar to start.
